# UBE2C at the crossroads of cell cycle regulation and DNA damage response in cancer

**DOI:** 10.1007/s10565-026-10230-y

**Published:** 2026-07-20

**Authors:** Sayandrila Paul, Amoolya Kandettu, Sanjiban Chakrabarty

**Affiliations:** Department of Public Health Genomics, Manipal School of Life Sciences, Manipal Academy of Higher Education, Manipal, India

**Keywords:** Ubiquitination, UBE2C, Cell cycle, DNA repair, Tumour progression, Cancer

## Abstract

The ubiquitin–proteasome system (UPS) is an important regulator of numerous cellular pathways, including DNA repair, protein degradation, the cell cycle, and signal transduction. UBE2C, a member of the ubiquitin-conjugating enzyme family, facilitates the ubiquitination of essential cellular regulators. Notably, UBE2C is consistently overexpressed in breast, lung, brain, hepatocellular, and ovarian carcinomas. Increased UBE2C expression is associated with tumour aggressiveness, genomic instability, epithelial-to-mesenchymal transition, and resistance to chemotherapy and radiotherapy. Our review explores the mechanistic involvement of UBE2C in diverse cancers, with a particular focus on its involvement in DNA damage repair mediated by the anaphase-promoting complex (APC/C). Furthermore, the regulatory effects of miR-381, miR-503, and miR-205 on UBE2C expression were examined, highlighting the promising prospects of these miRNAs for targeted cancer therapy. The involvement of UBE2C in different cancer hallmarks underscores its potential as a biomarker and therapeutic target. A comprehensive understanding of its mechanistic involvement in cell cycle regulation and DNA repair will further enhance its application in diagnostics, personalised medicine, and precision medicine.

**Graphical Abstract:**

UBE2C acts as a central regulator of tumour progression by coordinating cell-cycle control, oncogenic signalling, DNA damage repair, and therapy resistance. Dysregulated UBE2C expression promotes cancer cell proliferation, survival, genome maintenance, and treatment resistance, whereas its inhibition through miRNAs, RNAi, or pharmacological agents induces cell-cycle arrest, apoptosis, and enhanced therapeutic sensitivity, supporting UBE2C as an attractive target for cancer therapy. Created with BioRender.com.

**Figure:**
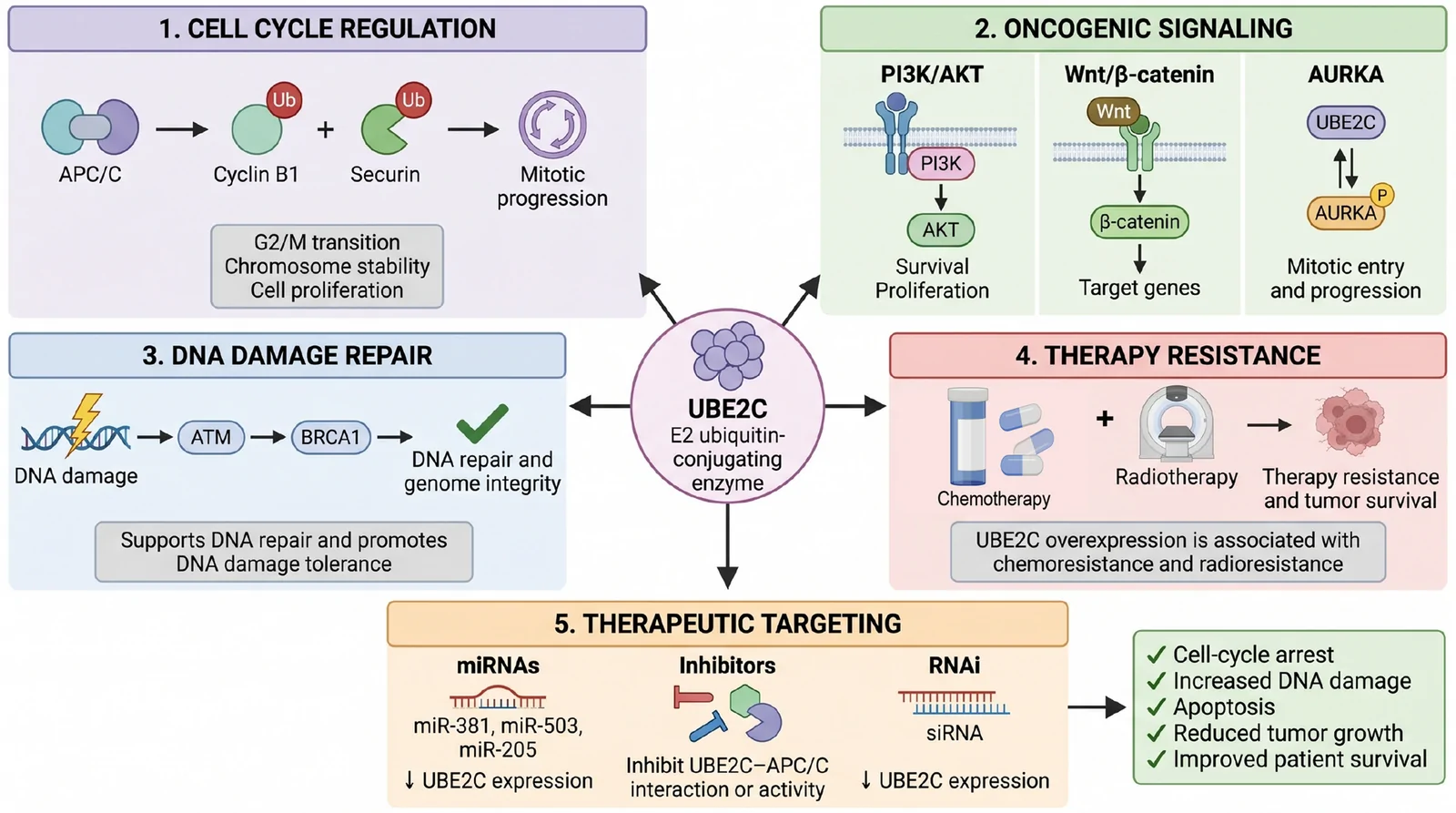

## Introduction

Ubiquitination is a fundamental process that regulates several cellular functions. This process is a crucial covalent modification of specific amino acid residues in a protein structure that determines the fate of the entire protein (Liao et al. 2024). Ubiquitination is a multistep process, mainly involving 1) ubiquitin activation via the formation of a thioester bond with a cysteine residue on the E1 enzyme, followed by 2) transfer of ubiquitin to the target protein by E2 enzymes, and 3) ligation of the active ubiquitin molecule on the target protein by E3 enzymes. This is followed by a downstream cascade. Ubiquitination is critical for maintaining protein turnover and ensuring the continuity of cellular processes via proteasomal proteolysis. This system is referred to as the ubiquitin–proteasome (UPS) system (Aliabadi et al.; Gupta et al. 2018). The UPS consists of specific enzymes that modify protein targets by attaching ubiquitin tags. If the UPS function is disrupted, it leads to an excess accumulation of malfunctioning proteins, which are critical for functions such as cell cycle, cell differentiation, DNA repair, and apoptosis (Awan et al. 2025). Dysregulation of the UPS has been implicated in neurodegenerative diseases and various cancers, highlighting its importance as a therapeutic and diagnostic target (McKeon et al. 2015). Among UPS components, UBE2C (ubiquitin‑conjugating enzyme E2 C), an E2 family member, has emerged as a key regulator of mitosis and cell‑cycle progression (Guo et al. 2018). UBE2C promotes the ubiquitination and degradation of mitotic cyclins, thereby controlling spindle microtubule dynamics and mitotic exit. Overexpression of UBE2C is associated with genomic instability and has been documented in multiple malignancies, including breast, ovarian, head and neck squamous cell carcinoma, and melanoma, where it correlates with aggressive tumour behaviour and poor prognosis. (Liu et al. 2020b).

Maintaining genome integrity requires coordinated regulation of cell cycle progression and DNA damage repair. Unrepaired DNA lesions can result in replication stress, chromosomal abnormalities, and accumulation of oncogenic mutations, ultimately driving tumour progression (Phillips 2018; Tubbs and Nussenzweig 2017).

Our review summarises the current evidence on the dual role of UBE2C in cell cycle regulation and the DNA damage response. We discuss how aberrant UBE2C expression contributes to tumour progression, therapy resistance, and genomic instability across various cancer types and highlight its potential as a prognostic biomarker and target for precision-oriented therapeutic strategies.

## The ubiquitin proteasome system (UPS)

**Fig. 1:**
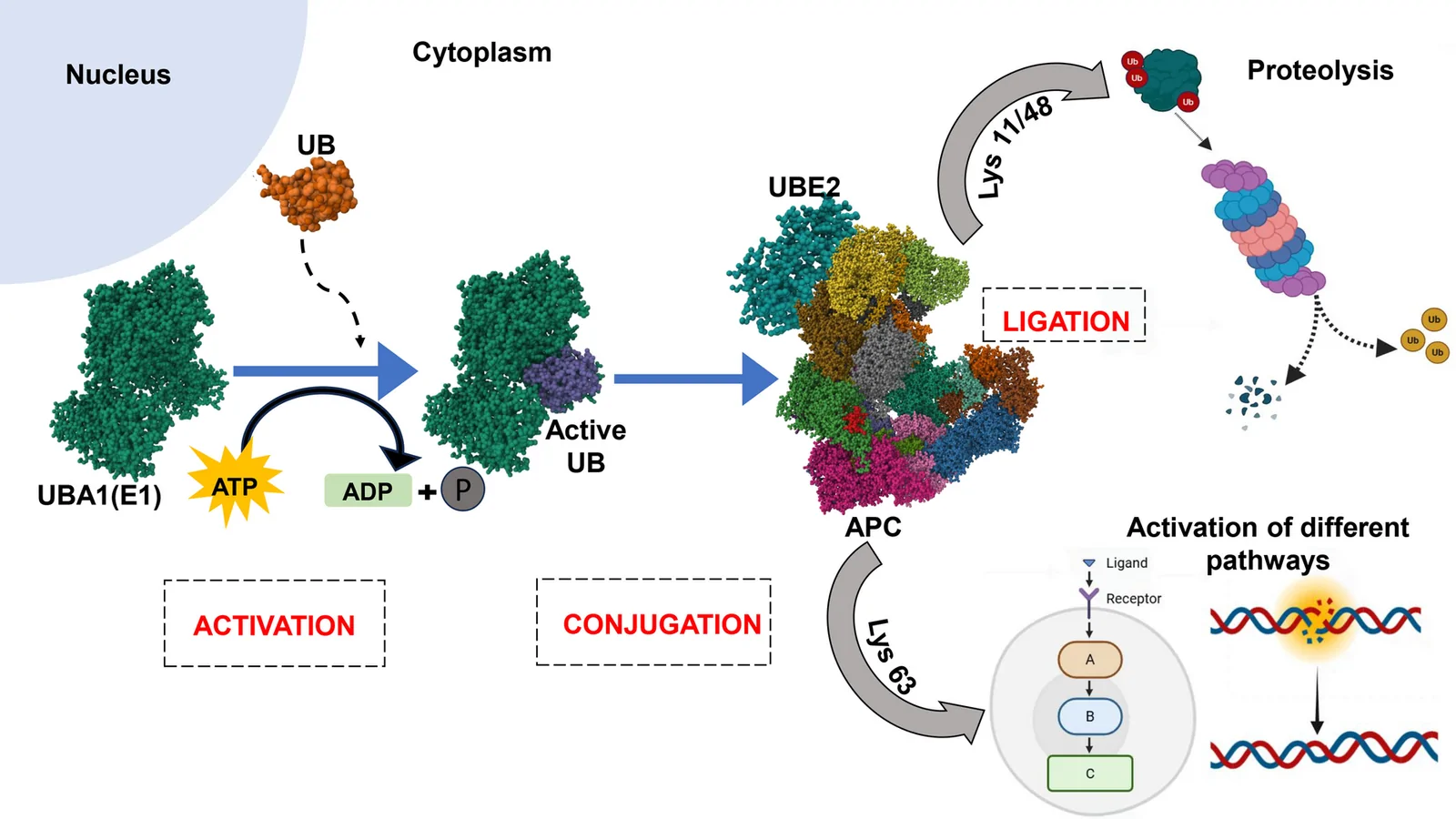
The ubiquitination cascade and cellular functions of ubiquitin signalling. Ubiquitination is initiated by activation of ubiquitin by the E1 enzyme UBA1, followed by transfer to E2 ubiquitin-conjugating enzymes and subsequent substrate modification through E3 ubiquitin ligases such as APC/C. Formation of distinct polyubiquitin chain linkages, including K11, K48, and K63, determines the fate of substrate proteins. While K11- and K48-linked chains predominantly mediate proteasomal degradation, K63-linked chains regulate signalling pathways, DNA repair, and other non-proteolytic cellular processes. Created with BioRender.com. rotein structures derived from (Brown et al. 2015; Chang et al. 2015; Lv et al. 2018)

The type of ubiquitination determines a protein’s fate, with most ubiquitin modifications serving as signals for proteasomal degradation. For instance, site-specific ubiquitination promotes protein phosphorylation, DNA damage repair signalling, and other signal transduction pathways (Awan et al. 2025; Waltho et al. 2024). Structurally, the 26S proteasome consists of one 20S core and one to two 19S regulatory subunits. The proteasome requires ATP for its function, which is responsible for protein hydrolysis. The 20S subunit is a barrel-shaped structure that contains the active catalytic site responsible for protein degradation. The 19S subunit selectively guides ubiquitinated proteins to the 20S subunit to facilitate deubiquitylation (Dikic 2017; Hao et al. 2012). Ubiquitination is initiated by the ATP-dependent activation of ubiquitin by the E1 enzyme, followed by conjugation through a trans-thiolation reaction. E2 enzymes collaborate with E3 ligases to transfer activated ubiquitin to lysine acceptor residues on substrate proteins. The specific ubiquitin linkage formed subsequently determines the fate of the modified protein (Fig. 1) (Waltho et al. 2024).

### The UBC(E2) enzyme family

**Fig. 2:**
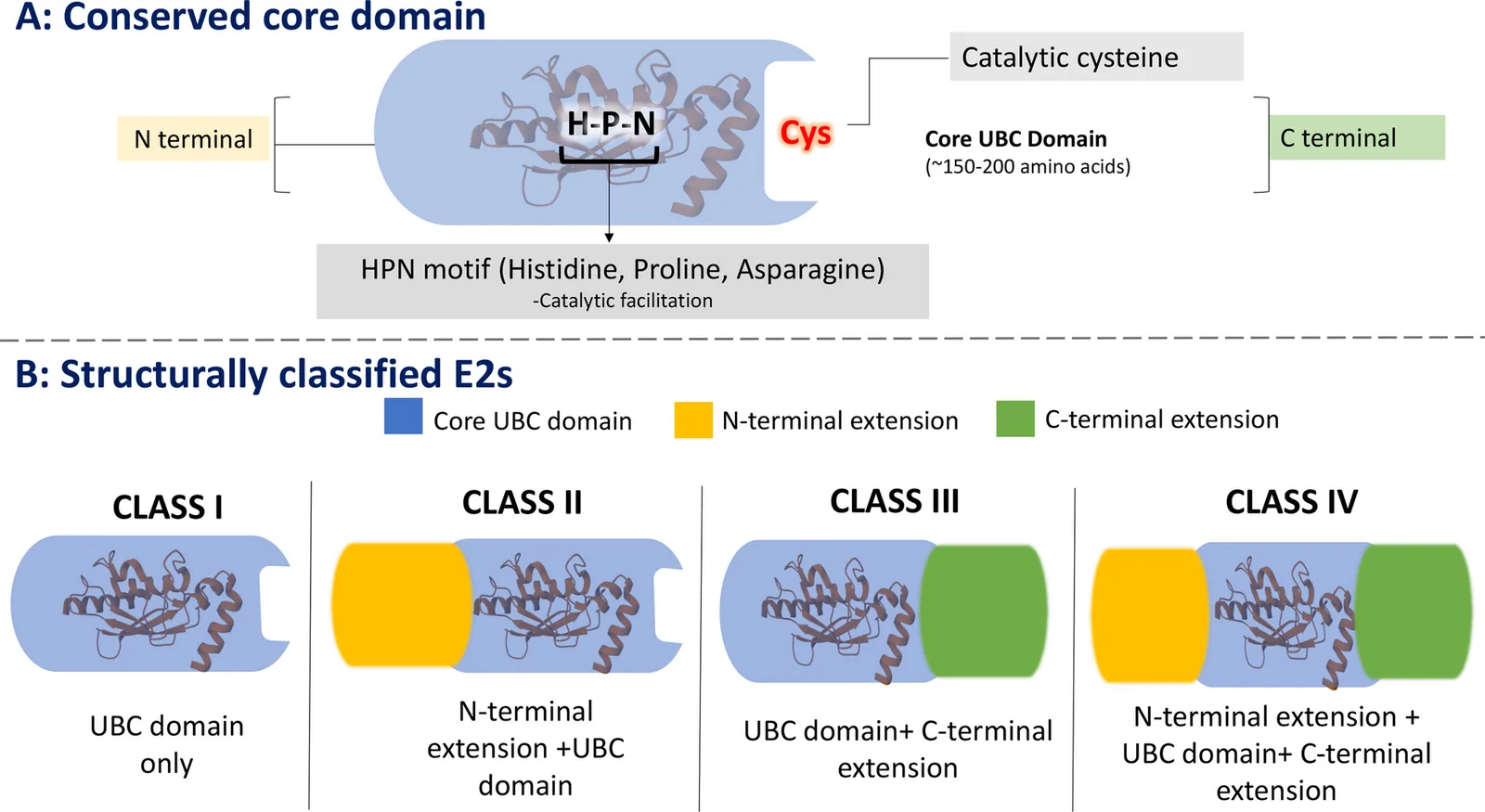
Structural basis and classification of E2 enzymes. **A**) Conserved core domain. Schematic representation of the highly conserved universal E2 core ubiquitin-conjugating domain (UBC), typically 150–200 amino acids in length. The diagram shows the relative positions of the catalytic cysteine (Cys) residue (highlighted in red) and the HPN motif (histidine, proline, and aspartic acid). Specific annotations point to the HPN motif’s role in catalytic facilitation. The peptide ends (N-terminal and C-terminal) are shown. **B**) Structurally classified E2s. Comparison of the four structural classes of E2 enzymes (I-IV), defined by the presence of terminal extensions relative to the core UBC domain. Class I, consisting solely of the UBC domain; Class II, possessing an N-terminal extension; Class III, featuring a C-terminal extension; and Class IV, possessing both N- and C-terminal extensions

Ubiquitin-conjugating enzymes (E2S or UBE2s) are core components of the UPS. These enzymes are conserved across all eukaryotes, with their encoding genes dispersed throughout the genome and showing no uniform organisational pattern. During evolution, the E2 family has expanded, culminating in 35 catalytically active E2 enzymes in humans. Differences in E2 repertoire size among organisms largely arise from gene duplication events (Ye and Rape 2009). In yeast, closely related E2 enzymes, such as UBC4 and UBC5, correspond to UbcH5A-D, UbcH6–H8, and UBE2E1 in higher eukaryotes. On the contrary, certain E2 enzymes are species-specific. For instance, the large E2 enzyme BIRC6 (Apollon) is absent in yeast but is conserved in humans, worms, mice, and flies. Likewise, UBE2W, HSPC105 (UBE2T), and Use1 (UBE2Z) are absent in yeast, whereas UBE2U appears to be restricted to mammals. All eukaryotes contain a conserved core set of E2 enzymes, which represent crucial components of the ubiquitination machinery, including UBE2M and UBE2I, despite their diversity. In higher organisms, additional E2S have emerged with specialised roles adapted to distinct cellular functions. E2 enzymes contain a conserved domain of approximately 150–200 amino acids, and the catalytic cysteine forms a transient thioester linkage with ubiquitin. This core domain exhibits around 35% structural similarity across species (van Wijk and Timmers 2010; Ye and Rape 2009). Nearly all UBC domains feature a conserved tripeptide having histidine, proline, and aspartic acid (HPN) positioned around the tenth residue preceding the Cys residue. Within the HPN, aspartic acid facilitates isopeptide bond formation, histidine is crucial for structural integrity, and proline stabilises the transition of these amino acids (Liu et al. 2020a). The classification of E2 enzymes is based on the location of an additional fragment within the UBC domain, resulting in four classes: Class I solely contains the UBC domain; Class II features the additional fragment present in both the N-terminal and UBC domains; Class III includes the fragment in both the C-terminal and UBC domains; and Class IV incorporates the fragment in the N and C terminals along with the UBC domain (van Wijk and Timmers 2010). All four classes contain a conserved core domain that includes a catalytic cysteine residue. The core of Class I requires an E3 enzyme to facilitate ubiquitin ligation, whereas classes II and III have extensions on the C- and N-termini of the core domain, respectively, that aid in recognising target proteins and regulating the ubiquitin conjugation process (Fig. 2). Specifically, class IV has both N-terminal and C-terminal extensions. The E2 proteins interact with specific E1 enzymes and, together with multiple E3 enzymes, function during ubiquitination (Ye and Rape 2009).

E3 ligases are broadly classified based on the presence of either a RING finger or HECT domain. The RING finger domain is defined by a conserved arrangement of cysteine and histidine residues that coordinate zinc ions in a cross-brace configuration. In contrast, the HECT domain comprises approximately 350 amino acids and adopts bilobed architecture, with the E2-binding site located in the N-terminal lobe and the catalytic cysteine residing in the C-terminal lobe. The lobes are connected by a flexible linker that forms a cleft, containing the active site (Yang et al. 2021). RING-type E3 ligases facilitate ubiquitin transfer by engaging activated ubiquitin-bound E2 enzymes and their substrates, whereas HECT E3 ligases directly accept ubiquitin from E2 onto their active-site cysteine prior to substrate conjugation. Irrespective of the distinct structural frameworks, both classes of E3 ligases rely on conserved interaction surfaces to engage E2 enzymes (van Wijk and Timmers 2010; Yang et al. 2021).

### UBE2C: an important regulatory E2 enzyme

**Fig. 3:**
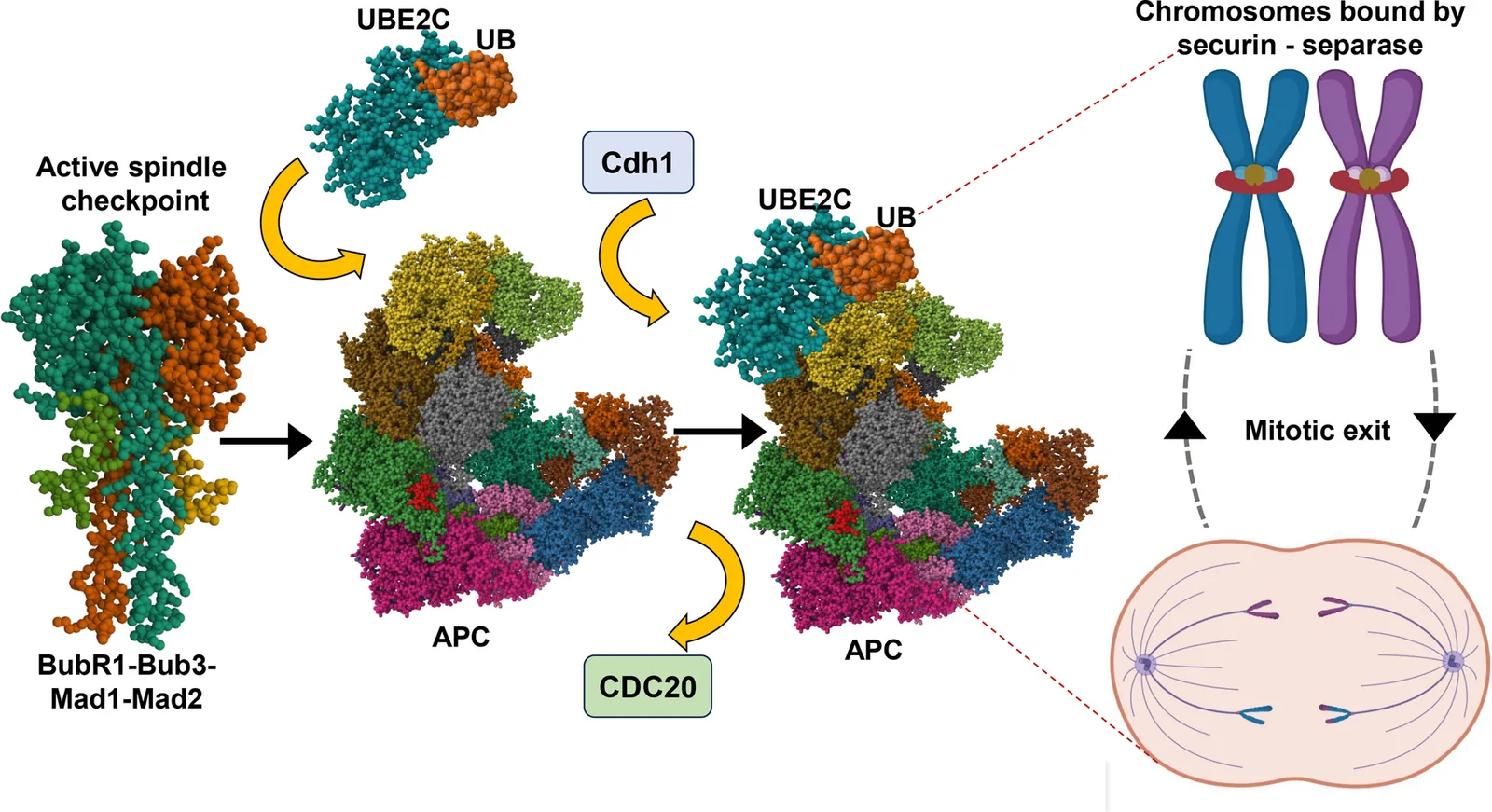
Regulation of mitotic exit by the UBE2C-APC/C ubiquitination axis. The spindle assembly checkpoint (SAC) restrains APC/C activity through the mitotic checkpoint complex (BubR1-Bub3-Mad2-CDC20) until proper chromosome attachment is achieved. UBE2C cooperates with APC/C to ubiquitinate mitotic substrates, leading to degradation of securin and cyclins, activation of separase, checkpoint silencing, and progression through mitotic exit. Subsequent APC/C-Cdh1 activity ensures orderly degradation of remaining mitotic regulators, thereby maintaining chromosome segregation fidelity and genomic stability. Created with BioRender.com. Protein structures derived from (Chang et al. 2015; Fischer et al. 2021; Lin et al. 2002; Lv et al. 2018)

UBE2C, a member of the ubiquitin-conjugating enzyme family (E2), is encoded by the UBE2C gene situated on human chromosome 20 (20q13.12). The UBE2C protein comprises 179 amino acids and has an approximate molecular mass of 20 kDa. It is also referred to as UbcH10 (Shen et al. 2013). This protein is localised in both the nucleus and the cytoplasm. It functions as a transferase, facilitating the transfer of ubiquitin from E1 to the target substrate protein (Zhan et al. 2024). For instance, UBE2C transfers ubiquitin from the E1 enzyme to the Apc11 RING subunit of the anaphase-promoting complex/cyclosome (APC/C), a key regulator of cell-cycle progression. This interaction enables the APC/C to function as an E3 ligase to target cyclins and components of the spindle assembly checkpoint (SAC), including budding uninhibited by benzimidazole 3 (Bub3), benzimidazole-related 1 (BubR1), mitotic arrest-deficient 1 (Mad1), and mitotic arrest-deficient 2 (Mad2), thereby ensuring proper mitotic progression (Wang et al. 2017). This inhibition of the spindle assembly checkpoint (SAC) is crucial for the progression of the cell cycle towards cytokinesis. The E2 enzyme contains a catalytic cysteine (Cys114) in its central domain, within the active site. This region facilitates the formation of a complex with E1-activated ubiquitin via a thiol ester bond. Subsequently, UBE2C, either in association with an E3 ligase or alone, transfers ubiquitin residues to specific target proteins, marking them for proteasomal degradation (Domentean et al. 2023). UBE2C contains a highly conserved N-terminal extension that regulates ubiquitin transfer and substrate lysine modification. This extended N-terminal region facilitates the ubiquitination of substrates that lack a canonical degron or defined ubiquitination signal. Fusion of the UBE2C N-terminal extension to UBE2D2, another E2 enzyme known to interact with the APC/C, does not impair ubiquitination activity, indicating that this segment functions compatibly within the established E2–E3 interaction framework. Collectively, these observations suggest an adaptive role for the N-terminal extension in fine-tuning APC/C-mediated ubiquitination (Liu et al. 2020b). The protein expression of UBE2C is regulated by the cell cycle. In the G1 phase, expression is at its lowest level, gradually increasing in the S phase and reaching its highest level in the G2 phase. The expression significantly decreases as the cells transition out of mitotic division (Fig. 3) (Xiang and Yan 2022).

In addition to UBE2C, the class II E2 enzyme family includes several additional members, such as UBE2E1 (UbcH6), UBE2E2, UBE2E3 (UbcH9), UBE2F, UBE2M (Ubc12), UBE2Q1, UBE2Q2, and UBE2V1. These enzymes participate in a wide range of ubiquitin-conjugation-dependent processes. Site-specific ubiquitination of target proteins governs diverse cellular pathways, including signal transduction, kinase activation, DNA repair, and proteasomal degradation, through distinct modes of modification, such as mono-ubiquitination, multi-mono-ubiquitination, and polyubiquitination. A growing body of evidence indicates that UBE2C is overexpressed across various cancer types, suggesting a potential oncogenic role warranting further mechanistic investigation (Jalali et al. 2024).

## Role of UBE2C in cancer

The role of UBE2C in human diseases, particularly cancer, has been extensively studied. These investigations have analysed DNA methylation patterns, and gene expression profiling datasets have consistently reported hypomethylation of the UBE2C gene promoter (Li et al. 2022). The involvement of UBE2C in cancer varies widely across tumour types; however, it primarily functions as a potent proto-oncogene that promotes uncontrolled cellular proliferation. Elevated UBE2C levels are directly correlated with increased cellular proliferation, chromosome instability, cellular invasion and migration, decreased apoptosis, suppression of autophagy, acquisition of stem-like characteristics, and dissemination and resistance to treatment. Mechanistically, many of these oncogenic effects are mediated by dysregulation of the spindle assembly checkpoint (SAC), aberrant APC/C signalling, activation of PI3K/AKT/mTOR and Wnt/β-catenin pathways, and suppression of p53-dependent tumour-suppressive signalling. Studies have shown that cells overexpressing UBE2C surpass the signals generated by the mitotic SAC, leading to loss of genomic stability, a characteristic cancer hallmark. Together, these factors contribute to unfavourable outcomes in patients with various malignancies, resulting in reduced survival rates and poor prognoses (Zhang et al. 2018a). UBE2C has also been revealed to be significantly hypomethylated in several cancers, including hepatocellular carcinoma (HCC), cholangiocarcinoma (CHOL), kidney renal clear cell carcinoma (KIRC), and pheochromocytoma & paraganglioma (PCPG) (Li et al. 2022, 2023; Zhang et al. 2018b). The role of UBE2C across different cancer types is discussed in Table 1.

**Table 1:** Poor disease progression is correlated with high expression of UBE2C in different cancer types

Cancer type	UBE2C expression	Impact of UBE2C	Reference
Brain	High	Tumour aggressiveness	(Ma et al. 2016)
Lung	High	Poor disease survival	(Zhao et al. 2012)
Ovary	High	Chemotherapy resistance	(Li et al. 2020)
Liver	High	Poor disease survival	(Ieta et al. 2007)
Oral	High	Tumour aggressiveness	(Jin et al. 2020)
Breast	High	Tumour aggressiveness	(Chou et al. 2014)

### Role of UBE2C in brain cancer

The involvement of UBE2C in brain tumours, including meningiomas, gliomas, and metastatic brain tumours, has been extensively investigated (Ma et al. 2016). In astrocytic tumours, elevated UBE2C expression has been reported in glioblastomas compared with lower-grade astrocytoma, indicating a positive association between UBE2C levels and glioma grade. In meningioma cells across different histological grades, UBE2C silencing suppresses cell proliferation, migration, and invasion, while inducing apoptosis, as evidenced by reduced Bcl-2 expression and increased Bax and caspase-3 levels. Moreover, malignant meningiomas exhibit marked downregulation of the tumour suppressor gene RIZ1, suggesting a c-Myc-dependent regulatory axis influencing UBE2C expression (Domentean et al. 2023). Furthermore, *in vivo* mouse experiments with UBE2C-overexpressing cancer cells revealed increased leptomeningeal dissemination, indicating an aggressive phenotype of brain metastatic disease. Another recent study employing single-cell RNA sequencing of human brain metastatic tissues from various cancer types identified UBE2C as a signature gene in the aggressive subgroup (Paisana et al. 2023). Studies have shown that increased levels of Aurora kinase B (AURKB) and UBE2C in glioma are associated with poor prognosis and resistance to temozolomide (TMZ) and radiotherapy. These findings suggest that targeting AURKB and UBE2C could offer potential therapeutic strategies and serve as prognostic indicators for evaluating the severity and long-term outcomes of glioma. The expression of AURKB and UBE2C in glioma, particularly glioblastoma (GBM), was assessed using data from the TCGA database, revealing significant upregulation compared to normal brain tissue (Alafate et al. 2019).

### Role of UBE2C in lung cancer

In lung adenocarcinoma, UBE2C has been shown to be critical for the proliferation of KRAS-mutant lung cancer cells and for the initiation of KRAS-driven lung tumorigenesis. During this process, UBE2C expression is upregulated, promoting the ubiquitination and subsequent degradation of the tumour suppressor DEPTOR through its interaction with the APC/C E3 ligase. These findings provide compelling evidence that the UBE2C/DEPTOR axis constitutes a regulatory cascade integrating oncogenic and tumour-suppressive signals in KRAS-induced lung tumour development (Zhang et al. 2023a). These findings establish the UBE2C–DEPTOR axis as an important regulatory mechanism that integrates oncogenic KRAS signalling with the suppression of tumour-inhibitory pathways. Further investigation is needed to explore the various networks involved. Elevated UBE2C levels are associated with advanced histological grade, gender, age, angiogenesis, postoperative survival duration, TNM stage, and unfavourable outcomes in non-small cell lung cancer (NSCLC) patients (Zhao et al. 2012). Mechanistically, UBE2C contributes to epithelial-to-mesenchymal transition (EMT), invasion, and metastasis through activation of PI3K/AKT and Wnt/β-catenin signalling pathways. Studies demonstrated that UBE2C knockdown decreases phosphorylated AKT, phosphorylated GSK-3β, β-catenin, and AURKA levels, indicating coordinated regulation of proliferative and EMT-associated signalling networks (Wang et al. 2017). Silencing UBE2C inhibits NSCLC cell proliferation and enhances chemotherapy sensitivity. Increased UBE2C expression may play a significant role in promoting the epithelial-to-mesenchymal transition (EMT), invasion, migration, and metastasis in lung cancer. UBE2C expression is correlated with the mutational status of p53 and EGFR, and this correlation can be further investigated to elucidate several networks (Wang et al. 2021a).

### Role of UBE2C in ovarian and uterine cancer

Elevated UBE2C expression has been linked to increased aggressiveness and chemoresistance in ovarian cancer. Notably, suppression of CDK1 leads to a concomitant reduction in UBE2C levels in ovarian cancer cells, indicating an upstream regulatory relationship in which CDK1 influences UBE2C expression, promoting tumour progression. Importantly, UBE2C knockdown effectively restores cisplatin sensitivity in cisplatin-resistant ovarian cancer cell lines, including SKOV3/DDP and A2780/DDP (Li et al. 2020). In endometrial cancer, accumulating evidence highlights the clinical and biological significance of UBE2C, which negatively regulates the p53 signalling pathway and thereby facilitates the epithelial-mesenchymal transition. Recent studies describing the estrogen-driven E2/ERα/UBE2C/p53 signalling axis further highlight its potential utility in endometrial cancer diagnosis and as a target for therapeutic intervention (Liu et al. 2020a). This signalling cascade contributes to enhanced proliferative capacity and metastatic potential in hormone-responsive tumours.

### Role of UBE2C in hepatocellular carcinoma (HCC)

Investigations into hepatocellular carcinoma (HCC) have reported that PRIM1 (DNA primase subunit 1) enhances the aggressive phenotype of HCC cells by regulating the AKT/mTOR signalling pathway and UBE2C-mediated inactivation of p53. The findings indicate that PRIM1 represents a promising therapeutic target, with potential relevance for both the prevention and treatment of HCC (Zhu et al. 2021). Increased UBE2C expression correlated with higher tumour grade in HCC patients and was associated with a poorer clinical prognosis. In vitro*,* UBE2C gene knockdown significantly reduced the invasion and proliferation in HCC cells. Studies have demonstrated the impact of UBE2C on the responsiveness of HCC cells to sorafenib treatment. This provides a platform to explore ubiquitination in HCC and to develop precise therapeutics (Ieta et al. 2007; Xiong et al. 2019).

### Role of UBE2C in oral squamous cell carcinoma (OSCC)

In oral squamous cell carcinoma (OSCC) cells, UBE2C suppression resulted in reduced cell proliferation, migration, invasion, and colony-forming capacity (Jin et al. 2020). This effect is accompanied by decreased expression of marker proteins for cancer stemness, including CD44, EpCAM, CD166, and ALDH1/2. Consistent with these findings, elevated UBE2C expression in OSCC tumour tissues correlates with poorer cellular differentiation and increased lymph node invasion (Liu et al. 2020b). In nasopharyngeal carcinoma, siRNA-mediated UBE2C silencing disrupted cell cycle progression in cancer cells. Treatment with bortezomib and the cell-cycle inhibitor CCI-779 further stabilises mitotic cyclins and impairs cell cycle dynamics by reducing UBE2C transcription and mRNA stability. Consequently, Downregulation of UBE2C exhibits decreased cell proliferation and induces cell cycle arrest at the S and G2/M phases. Collectively, these findings indicate that UBE2C represents a promising therapeutic target in nasopharyngeal carcinoma (Shen et al. 2013). These observations suggest that UBE2C promotes tumour progression primarily by regulating cell-cycle-associated signalling pathways and mitotic checkpoint control.

### Role of UBE2C in breast cancer (BC)

**Fig. 4:**
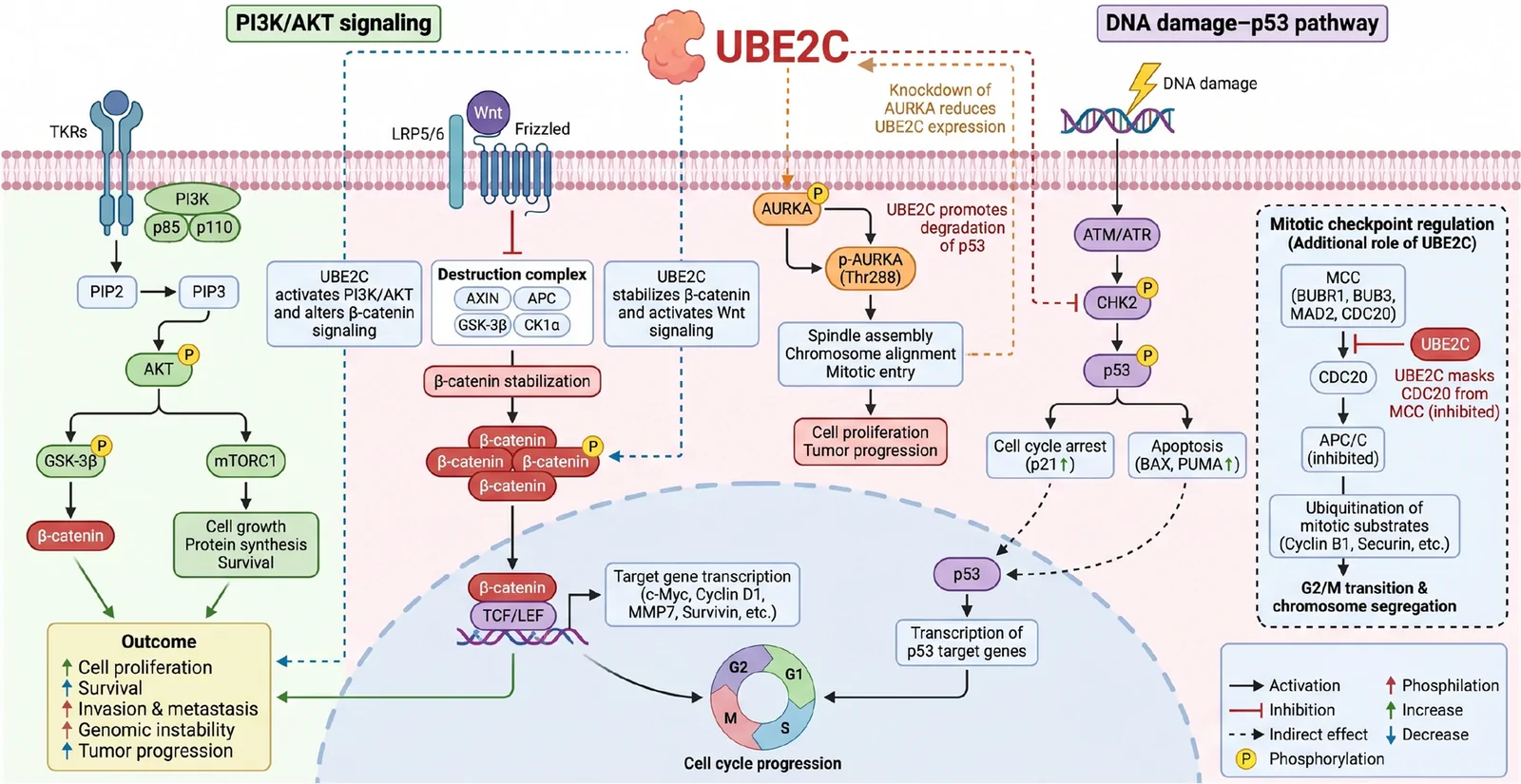
Major signalling pathways regulated by UBE2C in cancer. UBE2C promotes tumour progression by activating the PI3K/AKT and Wnt/β-catenin signalling pathways, thereby enhancing cell proliferation, survival, invasion, and metastasis. UBE2C-mediated activation of AURKA further stabilises β-catenin and stimulates transcription of oncogenic target genes, including MYC, CCND1, MMP7, and Survivin. In contrast, UBE2C suppresses the DNA damage response by promoting p53 degradation, thereby impairing cell-cycle arrest and apoptosis. Additionally, UBE2C masks CDC20 from the mitotic checkpoint complex (MCC), facilitating APC/C-mediated degradation of mitotic substrates and promoting G2/M transition and chromosome segregation. Collectively, these mechanisms establish UBE2C as a key regulator of oncogenic signalling, cell-cycle progression, and tumour development. Created with BioRender.com

In breast cancer (BC), UBE2C expression is distinctly upregulated in HER2-enriched and triple-negative breast cancer (TNBC) subtypes, and elevated UBE2C levels are strongly associated with disease progression, enhanced invasive potential, and poorer patient survival (Chou et al. 2014; Kariri et al. 2022). Comparative analyses of breast tumour tissues and adjacent normal counterparts revealed elevated UBE2C expression, accompanied by reduced NUMB levels, a cell fate determinant and endocytic adaptor protein, in tumour samples. The upregulation of UBE2C gene expression has been associated with disease progression, particularly with tumour grade and stage, whereas NUMB shows an inverse relationship (Guo et al. 2023). In hormone receptor-positive/HER2-negative (HR +/HER2–) breast cancer, elevated UBE2C expression led to promotion of estrogen-independent growth. Conversely, UBE2C downregulation suppresses cellular proliferation by disrupting cell-cycle progression in the presence of estrogen. (Kim et al. 2020). This process is closely linked to the ERα/UBE2C/p53 signalling axis, in which estrogen receptor signalling induces UBE2C expression, thereby attenuating p53-mediated apoptosis and cell-cycle arrest. Evaluation across multiple breast cancer cell lines identified MDA-MB-231 cells as exhibiting the highest basal UBE2C expression. In these cells, UBE2C knockdown led to upregulation of PTEN and marked suppression of phosphorylated mTOR, AKT, and HIF-1α, underscoring the critical role of UBE2C in regulating the mTOR/AKT signalling axis (Lu et al. 2021). By activating this pathway, UBE2C enhances proliferation, survival, metabolic adaptation, invasion, and potentially therapeutic resistance. Table 2 presents the key UBE2C-associated signalling pathways and the corresponding functional effects in cancer cells, including roles in proliferation, invasion, drug resistance, and therapy sensitisation (Fig. 4).

**Table 2:** UBE2C-associated signalling pathways and their functional outcomes in cancer

Tumour phenotype/biological process	UBE2C-associated signalling pathways	Key molecular mediators	Functional outcome in cancer cells	References
Cell proliferation	PI3K/AKT/mTOR pathway	PI3K, AKT, mTOR, Cyclin B1, CDK1	Promotes cell cycle progression, proliferation, and survival	(Lv et al. 2023; Tong et al. 2026)
Cell proliferation	Wnt/β-catenin signalling	β-catenin, c-Myc, Cyclin D1	Enhances proliferative signalling and tumour progression	(Wang et al. 2017; Wang et al. 2019)
Cell proliferation and mitotic progression	APC/C: UBE2C axis	APC/C, CDC20, Cyclin A/B, securin	Accelerates mitotic exit and weakens spindle checkpoint fidelity	(Domentean et al. 2023; Su et al. 2026; Yao et al. 2026)
Cell proliferation and genomic instability	FOXM1 signalling	FOXM1, PLK1, Cyclin B1	Drives mitotic gene expression and uncontrolled proliferation	(Liang et al. 2023; Lv et al. 2023; Shi et al. 2025a)
EMT and metastasis	MAPK/ERK signalling	MEK, ERK, MYBL2	Promotes EMT, migration, and invasion	(Kang et al. 2026; Long et al. 2023)
EMT and invasion	PI3K/AKT signalling	AKT, Snail, Twist, ZEB1	Facilitates EMT transition and metastatic dissemination	(Huang and Li 2024; Liu et al. 2020b; Wang et al. 2017; Xiong et al. 2019)
Drug resistance	BRCA1-associated DNA repair signalling	BRCA1, RAD51, HR proteins	Enhances DNA damage tolerance and resistance to chemotherapy	(Qin et al. 2017)
Drug resistance	ZEB1-ATM mediated	ATM, ATR, CHK1, ZEB1	Reduce genotoxic drug induced double strand DNA breaks	(Smith et al. 2024; Zhang et al. 2018a)
Drug resistance	ZEB1/2-mediated resistance pathways	ZEB1, ZEB2, ERCC1, ABCG2	Promotes chemoresistance and stem-like phenotypes	(Sakata et al. 2017; Shi et al. 2025b; Zhang et al. 2023a)
Drug resistance	TOP2A-associated pathway	TOP2A, mitotic regulators	Reduces sensitivity to anthracyclines and anti-mitotic drugs	(Yang et al. 2025)
Replication stress and DNA damage tolerance	APC/C–CtIP–53BP1 signalling	CtIP, 53BP1, APC/C	Alters HR/NHEJ pathway choice and DNA repair efficiency	(Escribano-Díaz et al. 2013)
Stemness and tumor adaptation	β-catenin and AKT-associated pathways	β-catenin, AKT, stemness regulators	Supports cancer stem cell maintenance and therapy escape	(Wang et al. 2017; Zhou et al. 2024)
Therapy sensitization	PARP inhibitor-associated synthetic lethality pathways	PARP, BRCA1, HR machinery	Increases vulnerability to DNA repair-targeted therapy	(Dibitetto et al. 2024; Ji et al. 2023; Rose et al. 2020)
Therapeutic targeting of UBE2C	PI3K/mTOR inhibition	Dactolisib, PI3K, mTOR	Downregulates UBE2C and suppresses metastatic progression	(Paisana et al. 2023)
Therapeutic targeting of UBE2C	MYBL2/UBE2C/MAPK axis	MYBL2, MEK, ERK	Suppresses EMT and invasive behavior in cancer	(Long et al. 2023)

## UBE2C and drug resistance

The majority of conventional chemotherapies include platinum and taxane-based drugs, which act through diverse mechanisms. Increasing evidence implies that UBE2C contributes to the development of drug resistance across multiple cancer types (Li et al. 2020). UBE2C overexpression in the TNBC cell line model showed increased resistance towards drugs such as doxorubicin, indicating its role in regulating drug response (Qin et al. 2017).

## UBE2C in DNA damage response

UBE2C plays a critical role in the spindle assembly checkpoint (SAC) by regulating the dynamic DNA damage response. As an essential E2 enzyme associated with the anaphase-promoting complex/cyclosome (APC/C), UBE2C facilitates SAC signalling by ubiquitinating cyclin B1, thereby promoting APC/C activation through its association with CDH1. An activated APC/C targets multiple substrates for ubiquitination, including C-terminal binding protein–interacting protein (CtIP). Through this regulatory cascade, UBE2C indirectly influences DNA damage repair by modulating homologous recombination (HR) and non-homologous end joining (NHEJ) pathways (Koo et al. 2023). The APC/C maintains genome integrity by regulating key factors of DNA damage response. Ubiquitination of 53BP1 promotes activation of homology-directed repair (HDR) at DNA damage sites. Accordingly, APC/C-mediated ubiquitination of DNA repair proteins is integral to coordinating damage-sensing and repair pathways (Zhu et al. 2021). A recent study on gastric cancer has revealed the role of UBE2C in DNA damage regulation and chemoresistance, particularly cisplatin therapy. UBE2C knockdown significantly impaired cell proliferation by inducing G2/M arrest and apoptosis. Additionally, the loss of UBE2C expression was associated with significant DNA damage, as evidenced by elevated levels of γ-H2AX and pATM and increased cisplatin sensitivity. The study identified MYBL2 as an upstream regulator of UBE2C. The overexpression of UBE2C reversed the effects of MYBL2 depletion and promoted cisplatin resistance, suggesting that the MYBL2/UBE2C axis mediated survival of gastric cancer cells from genotoxic stress and induced chemotherapy resistance (Long et al. 2023). A study by Yang et al., (2025) highlighted the role of UBE2C in regulating cellular response to DNA damage in breast cancer. UBE2C expression is significantly upregulated by FOXM1 transcriptional regulation. Inhibition of UBE2C reduced TOP2A levels through Parkin-mediated ubiquitination, leading to increased cellular senescence and enhanced doxorubicin sensitivity (Yang et al. 2025). UBE2C promotes TNBC progression by driving rapid cell-cycle progression and supporting tumour cell proliferation. Recent studies further indicate that UBE2C contributes to the cellular response to genotoxic stress by modulating DNA damage repair pathways. Elevated UBE2C expression has been linked to lower levels of DNA damage accumulation and decreased apoptosis following genomic damage. This suggests that it enables cancer cells to better withstand DNA damage and maintain survival under stressful conditions (Hu et al. 2025). Despite its importance, the precise mechanistic contribution of UBE2C to DNA repair remains incompletely characterised. Studies investigating the relationship between UBE2C and BRCA1 have demonstrated that UBE2C overexpression can downregulate BRCA1 and increase drug resistance, further supporting its role in DNA repair regulation and therapeutic response (Qin et al. 2017). Together, these data support a model in which UBE2C, via APC/C‑dependent ubiquitination, coordinates SAC function, cell‑cycle progression, and DNA‑repair pathway usage, thereby regulating genome integrity. Cancer cells with UBE2C overexpression exhibit increased proliferation and can circumvent senescence or apoptosis, even in the presence of DNA damage. Notably, breast cancer cell lines with reduced UBE2C expression exhibit increased sensitivity to radiation and chemotherapeutic agents, suggesting that UBE2C overexpression typically facilitates tumour cell survival under genotoxic stress conditions.

While UBE2C is not traditionally recognised as a core regulator of the ferroptosis pathway, several studies have suggested its indirect involvement in regulating ferroptosis (Cheng et al. 2026; Yang et al. 2026). Recent research has demonstrated that UBE2C-driven oncogenic signalling through the PI3K/AKT pathway may inhibit ferroptosis signalling (Wang et al. 2024). UBE2C interacts with p62 and KEAP1 to prevent NRF2 degradation. The absence of UBE2C impairs nuclear accumulation of NRF2 and reduces proliferation of gastric cancer cells (Jiang et al. 2024). It has been observed that downregulation of UBE2C in cancer cells increases proliferation, thereby augmenting the labile iron pool and reactive oxygen species (ROS), which contribute to ferroptosis (Wang et al. 2024). Further, in the context of cancer, this pathway promotes genomic instability and therapy resistance (Table 3).

**Table 3:** UBE2C-APC/C linked DDR regulators and functional outcomes

DDR node	Ubiquitination effect	Impact on HR/NHEJ	Functional consequence	References
Cyclin B1	APC/C—mediated degradation	Constrains HR window by driving mitotic exit	Promotes checkpoint recovery and DSB transmission if repair is incomplete	(Bassermann et al. 2008; Li and Zhang 2009)
CtIP	Ubiquitination and degradation	Reduces HR initiation and DNA-end resection	Favors NHEJ/MMEJ; may increase genomic instability	(Jeong et al. 2022; Lafranchi et al. 2014)
BRCA1	Downregulation linked to UBE2C overexpression	Impairs HR and HDR fidelity	Increases drug resistance and survival after DNA damage	(Dastsooz et al. 2019; Qin et al. 2017)
53BP1	APC/C—dependent ubiquitination	Promotes HDR factor recruitment	Enhances homology—directed repair at DSBs	(Bohgaki et al. 2013)

## Therapeutic targeting of UBE2C in cancer

The ubiquitin–proteasome system (UPS) establishes a regulatory network essential for maintaining protein homeostasis and its associated functions. UBE2C is intricately linked to cell cycle progression and cellular proliferation. Targeting this protein may facilitate tumour growth regulation by halting cellular proliferation, promoting genomic instability, and enhancing tumour cells' sensitivity to therapeutic agents. The spindle assembly checkpoint (SAC) maintains the mitotic cycle and regulates cell cycle intermediates, both of which are significantly influenced by UbcH10. The dysregulation of SAC is widely associated with the pathogenicity and disease progression (Lei et al. 2020). Therefore, targeting UBE2C could be an interesting area to explore for developing new treatment regimens for the associated diseases.

**Fig. 5:**
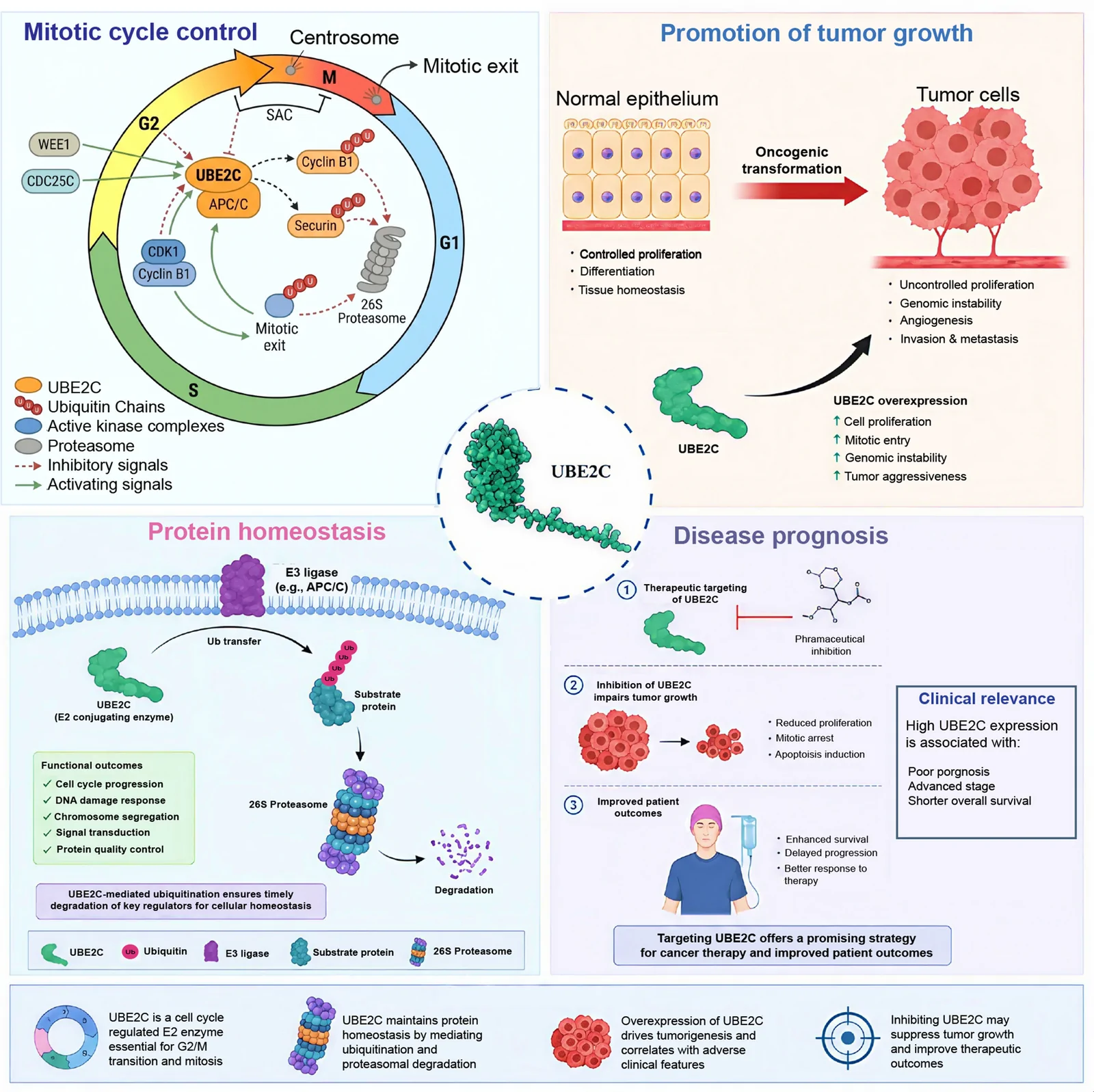
Multifaceted role of UBE2C in cell-cycle regulation, proteostasis, tumour progression, and therapeutic targeting. UBE2C, an E2 ubiquitin-conjugating enzyme, regulates cell-cycle progression through APC/C-mediated ubiquitination and degradation of key mitotic regulators, thereby ensuring proper G2/M transition and mitotic exit. UBE2C also contributes to cellular proteostasis by directing ubiquitin-dependent proteasomal degradation of target proteins involved in DNA repair, chromosome segregation, signal transduction, and protein quality control. Dysregulated UBE2C expression promotes tumour initiation and progression by enhancing proliferation, genomic instability, and metastatic potential. Elevated UBE2C levels are frequently associated with poor clinical outcomes, whereas therapeutic inhibition of UBE2C suppresses tumour growth and improves treatment response, supporting its potential as a prognostic biomarker and anticancer target. Created with BioRender.com

UBE2C is a promising therapeutic target in cancer research, as it plays a significant role in cell cycle regulation and is frequently overexpressed across multiple cancer types. The consistent upregulation in tumour tissues compared to adjacent normal tissues highlights its potential as a diagnostic marker and a therapeutic target. Experimental evidence shows that inhibition of UBE2C, either through gene silencing or pharmacological inhibition, induces cell cycle arrest, increases DNA damage, and sensitises cells to chemotherapeutic agents. For example, in breast and prostate cancer cell line models, knockdown of UBE2C conferred reduced clonogenic potential and increased sensitivity to doxorubicin and paclitaxel (Rawat et al. 2013; Wang et al. 2011). These findings suggest that UBE2C inhibition not only hampers the intrinsic tumorigenic capacity but can also overcome resistance to existing therapies. Therefore, targeting UBE2C could have a broader impact on tumour biology, extending beyond mitotic control, potentially affecting cancer stemness, immune evasion, and metabolic rewiring (Fig. 5).

Additionally, UBE2C expression levels are being explored as predictive biomarkers for treatment response and patient prognosis. High UBE2C expression often correlates with aggressive tumour behaviour, high histological grade, and poor overall survival, particularly in breast, lung, and liver cancers. Therefore, assessing UBE2C levels could aid in stratifying patients for targeted therapies and monitoring disease progression. Its potential utility in liquid biopsy-based detection has also been investigated, particularly in the context of circulating tumour DNA (ctDNA) and exosomal RNA profiling, which are gaining momentum in clinical oncology (Jalali et al. 2024).

Endogenously, gene regulation is controlled by a wide array of regulatory elements, including microRNAs, short non-coding RNAs. MicroRNAs (miRNAs) play a crucial role in post-transcriptional regulation, including targeting the UBE2C gene, which is implicated in various aspects of cancer progression. Numerous miRNAs have been identified as regulators of UBE2C expression in different cancer types. For example, miR-381, miR-503, miR-381-3p, and miR-381-5p have been shown to directly target UBE2C mRNA, leading to its downregulation and subsequent inhibition of cellular growth and invasion in various cancers, including breast, lung, and hepatocellular carcinoma. Similarly, miR-491-5p, miR-381, miR-140, and miR-300 have been reported to target UBE2C, thereby suppressing tumour growth and metastasis in lung, breast, and gastric cancer (Dai et al. 2021; Jiang et al. 2021; Wang et al. 2021a; Yu et al. 2021). Additionally, miR-205, miR-489, and miR-381 have been implicated in reversing drug resistance by targeting UBE2C in various cancers, including glioblastoma, pancreatic cancer, and cervical cancer. These findings emphasise the diverse roles of miRNAs in regulating UBE2C expression and their importance as therapeutic targets for cancer treatment. Further investigation into the specific miRNA-UBE2C regulatory networks could uncover novel avenues for developing targeted therapies and improving patient outcomes in cancer management (Li et al. 2023).

In renal cell carcinoma, long non-coding RNA (lncRNA) FTX has been found to function as an oncogenic regulator. The investigation reports that FTX acts as a competing endogenous RNA (ceRNA) by sponging miR-4429, preventing its binding to UBE2C and thereby upregulating UBE2C expression (Chen et al. 2022). In a study on oesophageal squamous cell carcinoma (ESCC) through RNA-sequencing data, tissue microarrays and immunohistochemistry, it was found that UBE2C is upregulated in ESCC. Further, the research focused on the construction of a ceRNA regulatory network, which showed a promising lncRNA HCP5-miR-139-5p and UBE2C network. Targeting this network demonstrated that HCP5 regulates UBE2C expression, thereby promoting ESCC progression (Li et al. 2021). In a pan-cancer study, a ceRNA regulatory network was developed to understand post-transcriptional regulation of UBE2C. The investigation reported multiple lncRNAs, emphasising NEAT1 and other miRNAs and circRNAs, highlighting the significance of exploring the regulation of UBE2C, which will impact cancer disease diagnosis, prognosis, and treatment (Jalali et al. 2024).

Research on UBE2C has advanced towards early drug development strategies and is now being tested in preclinical models. However, no UBE2C targeted agents have yet progressed to clinical trials in patients (Su et al. 2026). A first‑generation peptide‑based inhibitor of UBE2C has recently been designed using structure-guided approaches. This peptide competitively disrupts UBE2C-APC/C interactions and selectively suppresses proliferation and survival in multiple cancer cell lines, while sparing non‑transformed cells in preclinical models (Pirone et al. 2026). In addition to breast and prostate cancer, knockdown or pharmacologic inhibition of UBE2C has been shown to suppress tumour growth and enhance chemotherapy sensitivity in lung, colorectal, hepatocellular, ovarian, and gastric cancers in xenograft or organoid models. These studies consistently show cell‑cycle arrest, increased DNA damage, and reduced metastatic capacity upon UBE2C suppression (Dastsooz et al. 2019; Su et al. 2026; Zhao et al. 2024). UBE2C targeted strategies are particularly attractive in combination regimens because of its role in mitotic fidelity, DNA‑damage repair, and therapy resistance. Preclinical studies indicate that UBE2C depletion synergises with taxanes (e.g., paclitaxel) and DNA‑damaging agents (e.g., doxorubicin) by exacerbating mitotic catastrophe and replication stress (Su et al. 2026). Given that high UBE2C expression correlates with poor prognosis and chemoresistance, combining UBE2C-targeted agents with standard chemotherapy or targeted therapy could improve outcomes in patients with aggressive, UBE2C‑high tumours (Zhao et al. 2024). Overexpression of UBE2C in tumours versus adjacent normal tissue, and its association with advanced stage and high-grade tumours and poor survival in breast, lung, and liver cancers, support its use as a biomarker for selecting patients most likely to benefit from UBE2C‑directed therapies (Dastsooz et al. 2019; Jalali et al. 2024). Exploration of UBE2C levels in liquid biopsy platforms may enable non‑invasive monitoring of treatment response and early detection of resistance, although this remains largely experimental (Zhao et al. 2024). Currently, no prospective clinical trials targeting UBE2C have been reported. Clinical translation remains at an early stage, and existing evidence is largely confined to preclinical studies exploring UBE2C inhibition, degradation, or indirect suppression through upstream pathway targeting.

## Discussion

The overall protein turnover, function, and proteome dynamics are governed by ubiquitination. The UPS functions across the eukaryotes via a cascade of enzymes E1, E2, and E3 (Awan et al. 2025). In the E2 class of enzymes, UBE2C plays a crucial role in various cellular processes. UBE2C contributes towards tumorigenesis and is reported to show high expression in multiple types of cancer. Its impact extends to brain metastases, lung cancer, ovarian cancer, uterine cancer, hepatocellular carcinoma, oral cancer, and breast cancer, influencing cell proliferation, invasion, and resistance to therapy. Functionally, UBE2C facilitates protein ubiquitination, marking substrates for degradation and modulating signalling pathways crucial for cancer progression (Liu et al. 2020a; van Wijk and Timmers 2010). Under physiological conditions, UBE2C facilitates the degradation of cyclin B and securin. However, in cancer cells, UBE2C overexpression leads to premature degradation of cyclin B, bypass of the spindle checkpoint, and formation of supernumerary centrosomes, which, in turn, cause lagging chromosomes and aneuploidy (Ree et al. 2010). In the DNA damage response (DDR), UBE2C, together with UBE2S and RNF8, facilitates the formation of Lys11-linked ubiquitin chains on H2A/H2AX, thereby enforcing local transcriptional silencing (Paul and Wang 2017). Additionally, UBE2C ubiquitinates USP1 at sites of double-strand breaks, alleviating its suppression of BRCA1 and thereby promoting homologous recombination (Ha et al. 2017). Consequently, dysregulation of UBE2C permits cell-cycle re-entry with unrepaired lesions, biases repair pathway choice, and degrades checkpoint proteins, ultimately fostering mutagenesis and genomic instability. The loss of UBE2C induces G2/M arrest and apoptosis, highlighting its critical role in maintaining the balance between checkpoint and repair functions.

UBE2C expression has been associated with chemoradioresistance in cancer, presenting a significant challenge to current cancer treatment strategies and necessitating comprehensive investigation. These findings highlight the need to develop targeted approaches to overcome therapy resistance (Li et al. 2020). Targeting UBE2C holds potential to regulate tumour growth and sensitise cancer cells to treatment. While microRNAs (miRNAs) are emerging as epigenetic therapeutic targets that regulate UBE2C expression and influence cancer progression, understanding the complex regulatory networks involving miRNAs and UBE2C is crucial for developing innovative therapeutic strategies to manage cancer. Previously, multiple miRNAs (miR-525-5p, miR-381-3p, miR-196a, miR-300) have been identified that repress UBE2C expression in gastric, cervical, lung, prostate, and breast cancer (Han et al. 2015; Hu et al. 2019; Wang et al. 2021a, 2021b). UBE2C has been shown to suppress apoptosis (Hu et al. 2025).

Another study reported that inhibition of UBE2C increased the labile iron pool and ROS, promoting ferroptosis in AML cells, thereby widening the scope for exploring UBE2C's role in ferroptosis and its significance in cancer therapy (Wang et al. 2024). It has been reported that wild-type p53 suppresses UBE2C transcription through a p21-E2F4 complex, whereas mutant p53 can activate UBE2C (Bajaj et al. 2016). Increased UBE2C levels facilitate epithelial-mesenchymal transition (EMT) and chemotherapy resistance by ubiquitinating EMT effectors, such as vimentin, which helps maintain the mesenchymal state (Liu et al. 2020a; Pan et al. 2021; Wang et al. 2019). UBE2C enhances the Wnt/β-catenin and PI3K/Akt–Aurora A signalling pathways, leading to the suppression of E-cadherin and an increase in N-cadherin expression (Wang et al. 2017). Clinically, higher UBE2C expression is linked to enhanced cell migration, invasion, and drug resistance. The synthesis of these findings proposes a model where the absence of anti-UBE2C miRNAs or p53 inactivation leads to increased UBE2C levels. Consequently, UBE2C degrades DEPTOR, activating mTORC, and suppresses epithelial markers via the Wnt/PI3K-EMT pathways, ultimately fostering chemoresistance and metastasis (Zhang et al. 2023b).

Investigating the role of UBE2C in tumorigenesis provides a platform for exploring cancer biology and offers opportunities for developing targeted therapies to combat cancer progression and improve patient outcomes. Understanding the mechanisms that affect UBE2C function and its interactions with miRNAs is essential for developing novel therapeutic approaches for cancer treatment. The development of pharmacological inhibitors of UBE2C is still in its early stages; the continued integration of genomic, proteomic, and structural biology tools is likely to accelerate the discovery of effective UBE2C-targeted therapies. Approaches combining UBE2C inhibition alongside standard chemotherapeutics or pathway-specific inhibitors may also offer synergistic benefits and help overcome drug resistance (Jalali et al. 2024). As our understanding of UBE2C's role in cancer deepens, it promises to be not only a therapeutic target but also a biomarker for personalised oncology strategies.

Research on UPS has attracted an increasing interest over time. However, there is less clarity about the involvement of UPS components, such as UBE2C, in DNA damage repair pathways and about how they affect normal physiological conditions. Insights into DNA damage repair pathways and ubiquitination will significantly contribute to understanding the mechanisms a cancer cell employs to survive, combat, and overcome therapy. There are no reported clinical trials for UBE2C to date, but the ongoing studies are interestingly expanding the scope for investigating UBE2C as a potential target and developing targeted therapy (Cai et al. 2024). The importance of targeting UBE2C and its effects on disease progression are among the questions that remain to be explored and understood to develop better diagnostics and targeted therapies (Koo et al. 2023; Petropoulos et al. 2019; Tubbs and Nussenzweig 2017).

Interestingly, even with the growing evidence for UBE2C as an oncogenic driver and a potential therapeutic target, several challenges and key gaps remain. The mechanisms by which UBE2C contributes to therapy resistance, particularly in the context of taxanes, DNA-damaging agents, and emerging targeted therapies, remain incompletely defined. While UBE2C overexpression is linked to genomic instability, mitotic stress, and impaired DNA damage response signalling, it is unclear if the resistance arises primarily from mitotic bypass, altered checkpoint control, or rewiring of ubiquitin‑dependent repair pathways (Sazonova et al. 2021; Su et al. 2026). UBE2C’s functions appear to be highly context dependent across tumour types, yet most mechanistic studies are limited to a few cancers (e.g., breast, prostate, liver, and brain tumours). The precise roles of UBE2C in other malignancies remain poorly characterised. This hinders the development of tumour-selective intervention strategies (Cai et al. 2024; Lu et al. 2021; Paisana et al. 2023; Wang et al. 2011). The lack of clinically approved UBE2C inhibitors currently limits the translation of preclinical findings into patient-oriented trials, and the potential for on‑target toxicity in normal proliferating tissues remains a concern. Integrating UBE2C expression into multimodal biomarker panels, alongside genetic drivers, immune‑signature profiles, and ctDNA‑based monitoring, will be essential to determine which patient subsets are most likely to benefit from UBE2C targeted therapies. It is crucial to understand the full potential of UBE2C in precision oncology by addressing these gaps through systematic functional studies, cancer-type-specific models, and biomarker-driven preclinical trials (Alafate et al. 2019; Su et al. 2026). Future research should therefore focus on defining UBE2C-dependent resistance networks, elucidating its tissue‑specific functions, and developing safe and effective UBE2C-targeting agents suitable for clinical translation.

## Data Availability

No datasets were generated or analysed during the current study.
